# Acute effects of anti‐seizure medications on network level dynamics: A systematic comparison using multielectrode arrays

**DOI:** 10.1002/epi.70408

**Published:** 2026-08-01

**Authors:** Griselda Marku, Lena Gschossmann, Kerstin Hallmann, Heinz Beck, Kunihiko Araki

**Affiliations:** ^1^ Institute of Experimental Epileptology and Cognition Research University of Bonn, Faculty of Medicine Bonn Germany; ^2^ University Hospital Bonn Bonn Germany

**Keywords:** anti‐seizure medication (ASM), multi‐electrode array (MEA), multivariate clustering analysis, neuronal network

## Abstract

**Objective:**

A multi‐electrode array (MEA) is a powerful extracellular recording technique for long‐term monitoring of neuronal network activity. In recent years, MEA‐based assays have been applied increasingly to evaluate drug efficacy and neurotoxicity, including the assessment of anti‐seizure medications (ASMs). However, systematic comparisons of ASMs with different mechanisms of action under identical experimental conditions remain limited.

**Methods:**

In this study, we evaluated the effects of 18 ASMs on neuronal network activity using MEA recordings under standardized conditions. Network activity was characterized using four components—spike, burst, network burst, and synchrony—and a total of 44 MEA‐derived parameters were analyzed. Dimensionality reduction by t‐distributed stochastic neighbor embedding (t‐SNE) followed by hierarchical clustering classified the 18 ASMs into four distinct clusters.

**Results:**

Cluster 1, including S‐licarbazepine, oxcarbazepine, and lamotrigine, exhibited pronounced effects across all four network components. Cluster 2, comprising carbamazepine, eslicarbazepine acetate, phenytoin, phenobarbital, and stiripentol, significantly affected spike and burst duration but showed only limited effects on burst frequency, network burst frequency, or synchrony. Cluster 3 was solely diazepam, for which minimal effects were detected. The remaining ASMs were assigned to Cluster 4 and produced only minimal measurable effects in network activity.

**Significance:**

Several limitations should be considered, including the use of primary neuron cultures, incomplete maturation of certain drug targets, evaluation of only acute drug effects, and the limited spatial resolution of low‐density MEA systems. Nevertheless, our results demonstrate that this MEA‐based platform enables systematic evaluation of ASM effects on neuronal networks under identical experimental conditions. Furthermore, multivariate and unsupervised clustering analysis of 44 MEA parameters facilitated classification independent of drug mechanism of action. These findings suggest that MEA, combined with multidimensional data analysis, may provide a useful platform for the direct comparison of ASMs and could contribute to future pharmacological screening and drug discovery efforts.

**Key points**

- We were able to classify the 18 anti‐seizure medications (ASMs) into four distinct clusters based on the pharmacological effect profile.
- Cluster 1 (S‐licarbazepine, oxcarbazepine, lamotrigine), exerted the broadest effects, consistently modulating single cell firing and network‐based parameters.
- Cluster 2 (carbamazepine, eslicarbazepine, phenytoin, phenobarbital, stiripentol) primarily influenced spike and burst duration, with little to no effect on burst frequency or network burst, or synchrony.
- Diazepam, the sole member of Cluster 3, produced only limited effects on neuronal network.
- Cluster 4 (the remainder of ASMs) showed minimal measurable effects.

## 1 INTRODUCTION

Epilepsy is a chronic neurological disorder characterized by recurrent seizures resulting from excessive and hypersynchronous neuronal firing.^1^ Anti‐seizure medications (ASMs) remain the mainstay of therapy, with many exerting their effects primarily through modulation of voltage‐gated sodium channels (VGSCs) or enhancement of γ‐aminobutyric acid (GABA)–mediated inhibitory neurotransmission.^2^ Over recent decades, the therapeutic landscape has expanded to include ASMs with diverse, and often multimodal mechanisms of action, resulting in ~30 clinically available agents.^2^ Despite this progress, seizure control remains inadequate in nearly 30% of patients,^3^ highlighting a critical need to better understand ASM actions and for the development of therapies targeting mechanism underlying drug‐resistant epilepsy.^4^

In vitro models provided an important platform for evaluating the pharmacological effects of ASMs under controlled experimental conditions.^5^ Such studies include acute brain slices, whole‐brain preparations, organotypic slice cultures, isolated cells, and dissociated cell cultures, each offering distinct advantages and limitations. Dissociated primary cortical neuron cultures form functional synaptic networks that generate spontaneous neuronal activity and are therefore well suited for investigations using multi‐electrode array (MEA) technology.^5^

MEA technology enables simultaneous, long‐term recordings of population‐level neuronal activity with high throughput and excellent experimental reproducibility. Consequently, MEA‐based assays have become increasingly important for drug screening, neurotoxicity testing,^6^, ^7^ and the functional evaluation of ASMs.^8^ Although numerous studies have investigated individual compounds, this technology has not been used systematically across ASMs of different classes. Such a standardized framework would enable direct comparison of drug‐induced network signatures independently of their conventional pharmacological classification.

In the present study, we therefore systematically evaluated the acute effects of 18 clinically used ASMs under identical conditions to establish a comparative, network‐level pharmacological profile. A comprehensive set of MEA‐derived parameters was analyzed using hierarchical clustering, resulting in four clusters of ASMs. To facilitate cross‐compound comparison, effect profiles were visualized using radar plots, enabling the identification of distinct and reproducible patterns of network modulation associated with different mechanisms of action.

## 2 MATERIALS AND METHODS

### 2.1 Primary cortical neuron culture

All animal procedures were planned and performed to minimize pain and suffering, and to reduce the number of animals used in accordance with European, national, and institutional guidelines (guidelines of the European Parliament and of the Council on the protection of animals used for scientific purposes, European Directive (2010/63/EU), and the ARRIVE guidelines (Animal Research: Reporting of In Vivo Experiments)). The study protocol was approved by the Landesamt für Natur, Umwelt und Verbraucherschutz (LANUV) of the state of North Rhine‐Westphalia, Germany. Pregnant female mice (C57BL/N) were obtained from Charles River Laboratories. For each experiment, a single pregnant mouse constituted one biological preparation. Between 3 and 11 embryos (E17) were obtained from each pregnant mouse under deep isoflurane anesthesia and transferred to a 10‐cm Petri dish on ice. Following decapitation, brains were dissected under a stereomicroscope at room temperature. Cortices from all embryos originating from the same pregnant mouse were pooled and processed together, generating one primary cortical culture preparation per animal. The pooled cortical tissue was transferred to 1.8 mL Hank's balanced salt solution (HBSS (−), 14 170–088, 500 mL, Gibco) and 200 μL trypsin (15 090 046, 2.5% trypsin, 100 mL, Gibco), and then incubated at 37°C and 5% CO_2_ for 15–20 min. The cortical tissues were triturated with 1 mL of 10 mg/mL DNase I (final concentration 1 mg/mL) (11284932001, 100 mg, Roche) and 120 μL of 1 M MgSO_4_ · 7H_2_O and 9 mL of cold HBSS (−). The triturated tissue was washed with Eagle's basal medium (BME) (41010‐026, 500 mL, Gibco) complemented with 2% B27 (17504‐044, 10 mL, Gibco), 2.4% L‐glutamine (25030‐024, 1.2 mL, Invitrogen, Gibco), 1% fetal bovine serum (FBS, 5 mL), and 1% glucose (G8769, 5 mL, Sigma). Depending on the number of embryos obtained, each biological preparation yielded between 5.67 and 14.3 million cells and was used to seed ~3–5 independent 24‐well MEA plates (CytoView MEA 24, M384‐tMEA‐24 W, Axion Biosystems). The cell suspension containing 40 000 neurons per 5–20 μL was plated in one well on a 24‐well MEA plate (CytoView MEA 24, M384‐tMEA‐24 W, Axion Biosystems) and incubated at 37°C and 5% CO_2_ for 30–60 min. Plating was carried out with identical BME supplemented with B27, L‐glutamine, FBS, and glucose (700 μL/well).

### 2.2 MEA recordings

#### 2.2.1 MEA settings

MEA recordings were conducted using a Maestro Edge System with CytoView‐MEA24 well plates from Axion Biosystems (M384‐tMEA‐24 W, 4 × 4 grids [1.1 mm × 1.1 mm] = 16 electrodes, 1 grid = 50 μm diameter). Neurons were cultured on MEA 24‐well plates and recorded every 2–3 days. A baseline recording was initially carried out in the absence of ASMs, followed by experiments to observe the acute effects of ASMs at DIV 15–25. The Maestro settings are as follows: a sampling frequency of 12.5 kHz, a gain of 1000×, and a band‐pass filter of 200–3000 Hz served as the default settings. For MEA recordings, the Maestro system was maintained at 37°C and 5% CO_2_. The 24‐well MEA plate containing the cultured primary cortical neurons was placed into a docking bay and allowed to equilibrate for 5 min.

Each experiment consisted of three phases: baseline, wash‐in, and recovery. First, spontaneous activity was recorded for 5 min under control conditions (baseline). ASMs were then applied, and neuronal activity was recorded for an additional 10 min (wash‐in). To allow sufficient time for drug effects to stabilize, only the final 5 min of the wash‐in period were used for subsequent analyses. Following drug exposure, ~80% of the culture medium was replaced with fresh BME (700 μL per well supplemented with 2% B27, 2.4% L‐glutamine, 1% FBS, and 1% glucose). Preliminary experiments demonstrated that immediate recordings after medium exchange resulted in transient increases in neuronal activity, likely due to mechanical disturbance and changes in the extracellular environment. Therefore, recovery recordings were performed 20–24 h after medium replacement. These measurements are presented to illustrate the evolution of network activity following drug removal under the experimental conditions and should not be interpreted as evidence of complete or immediate reversibility of ASM effects.

#### 2.2.2 MEA data analysis

The experimental unit was defined as a single MEA well. Multiple wells and plates derived from the same pooled embryo preparation constituted technical replicates originating from one biological preparation (one pregnant mouse). Because individual ASMs were not replicated systematically across independent biological preparations, the present analyses should be interpreted as comparative pharmacological signatures obtained under standardized assay conditions rather than estimates of between‐animal variability.

The raw data (RAW files) were analyzed by Axion Integrated Studio (AxIS) software (Axion BioSystems; version 3.7.3), and the spike files (SPK files) were analyzed by the NeuralMetric Tool (Axion BioSystems; version 4.0.5). This AxIS navigator identifies spikes and bursts using previously described spike and burst detectors. Forty‐four activities were grouped broadly into four categories: spiking, single‐electrode bursting, network bursting, and synchrony. The mean firing rate for spiking, burst duration, and frequency average for single‐electrode bursting, network burst frequency average, and synchrony index were selected for evaluation, with results showing good reproducibility from the baseline measurements without drugs. Spikes were detected when the signal crossed the threshold of 6× standard deviation (SD) of the baseline. The mean firing rate indicates the frequency of action potentials produced by neurons. A cluster of spikes (5 spikes less than 100 ms) was identified as a burst, and when it was coordinated across multiple electrodes, it was termed a network burst, representative of synaptic communication between neurons. The synchrony index is a unitless measure of synchrony in the range 0 to 1^9^ that reflects the influence of functional synapses between neurons, and was quantified by the AxIS software using a cross‐correlogram. Due to limitations in accurately counting spikes at constructive and destructive interference, spike metrics should be interpreted in conjunction with the corresponding burst and network synchrony data.

In a first step, outliers defined as values more than 3 SD from the mean were removed. To compare baseline and wash‐in values for every parameter and ASM, activities were normalized to the activities in the presence of dimethyl sulfoxide (DMSO) in the same plate.

$$\text{Normalized baseline activity}=\frac{\text{Activity}\;\left(\text{Baseline}\right)\;}{\text{Averaged activity}\;\left(\text{DMSO}\right)}$$

$$\text{Normalized wash}\_\text{in activity}=\frac{\text{Activity}\;\left(\text{Wash}\_\text{in}\right)\;}{\text{Averaged activity}\;\left(\text{DMSO}\right)}$$

This accounted for the potential DMSO toxicity and activation due to the wash‐in. In addition, the effects of ASMs were evaluated by normalizing each sample to its baseline before drug wash‐in.

$$\text{Remaining activity}=\frac{\text{Normalized wash}\_\text{in activity}\;}{\text{Normalized baseline activity}}$$

The effect size of ASM is the change in activity, determined by subtracting the remaining activity from 100%, with each parameter represented as one axis on the radar chart.

$$\mathrm{ASM}\;\text{effect size}=\left(1-\text{remaining activity}\right)\times 100\;\left(\%\right)$$

MEA activity is susceptible to drying and human intervention, such as medium exchanges, and can easily change. Therefore, standardization with DMSO on the same plate and confirmation of activity recovery after wash‐out can more accurately evaluate its effectiveness.

### 2.3 Drugs and reagents

All 18 drugs were prepared freshly. Carbamazepine (CBZ, C4024, Sigma‐Aldrich, 25 μM, 50 μM), oxcarbazepine (OXC, PHR 1635, Merck, 25 μM, 50 μM), eslicarbazepine acetate (ESL, B5435, Merck, 25 μM, 50 μM), S‐licarbazepine (S‐Lic, 35 008, Cayman chemicals, 50 μM, 250 μM), lacosamide (LCM, Y0001982, Merck, 50 μM, 100 μM), phenytoin (PHT, PHR1139, Merck, 50 μM, 100 μM), cenobamate (CBM, DC60042, DC Chemicals, 50 μM, 100 μM), lamotrigine (LTG, PHR1392, Merck, 25 μM, 100 μM), stiripentol (STP, S5266, Selleckchem, 50 μM, 100 μM), rufinamide (RFM, R8404, Merck, 50 μM, 100 μM), topiramate (TPM, T0575, Sigma‐Aldrich, 25 μM, 100 μM), phenobarbital (PB, Neuraxpharma, 25 μM, 50 μM), and levetiracetam (LEV, OHR1447, Merck, 100 μM, 300 μM) were dissolved in DMSO at a concentration of 100 mM. A 100 mM solution was diluted 10‐fold with medium (to 10 mM), and 1.8 μL (25 μM), 3.5 μL (50 μM), 7 μL (100 μM), 17.5 μL (250 μM), and 21 μL (300 μM) were dispensed into wells containing 700 μL each. Brivaracetam (BRV, 29X.03, Carl Roth, 10 μM, 50 μM) was dissolved in DMSO at a concentration of 50 mM. A 50 mM solution was diluted 10‐fold with medium (to 5 mM), and 1.4 μL (10 μM) and 7 μL (50 μM) were dispensed into wells containing 700 μL each. Fenfluramine (FFA, axon2850, Axon Medchem, 5 μM, 20 μM) was dissolved in DMSO at a concentration of 1 mM. A 1 mM solution was diluted 100‐fold with medium (to 1 mM), and 7 μL (5 μM) and 28 μL (20 μM) were dispensed into wells containing 700 μL each. Cannabidiol (CBD, C6395, Sigma‐Aldrich, 2.5 μM, 5 μM) was 1 mg/mL (3.18 mM), 2.5 μL (2.5 μM) and 11 μL (5 μM) was dispensed into wells containing 700 μL each. Diazepam (DZP, Domespharma, .3 μM, 1 μM) was 5 mg/mL (17.55 mM); 1.3 μL (.3 μM) and 3.9 μL (1 μM) were dispensed into wells containing 700 μL each. Control medium included equal concentrations of DMSO, which was finally diluted to less than one part per thousand. Valproic acid (VPA, P4543, Merck, .5 mM, 1 mM) was dissolved in BME medium at a concentration of 100 mM. 3.5 μL (.5 mM) and 7 μL (1 mM) at 100 mM were dispensed into wells containing 700 μL each. For VPA, control medium did not include DMSO. For phenobarbital, the tablet was crushed, and the resulting powder was weighed and used.

### 2.4 Data analysis (pairwise Pearson correlation, principal component analysis [PCA], hierarchical clustering, t‐distributed stochastic neighbor embedding [t‐SNE])

Raw data are provided in Table S1. Data analyses were performed in Python (version 3.12.7) using the Scikit‐learn (1.9.0) and Scipy (1.17.1) libraries. For all analyses described in the following section, effect size averages across samples for each parameter and ASM were used, including only the high‐concentration condition for each drug. Interdependence of parameters was assessed using the Pearson correlation coefficient among pairs of parameters. In addition, a PCA (16 components) was performed to inspect the contribution of individual parameters to the overall variation among ASMs. To group ASMs by their effect on five selected parameters—mean firing rate, burst duration, burst frequency, network burst frequency, and synchrony index—we performed hierarchical clustering on z‐scored values. The distance between clusters was computed using the unweighted pair group method with arithmetic mean (UPGMA) re algorithm and the optimal number of clusters was determined as the number yielding the highest silhouette score within the range of 3 to 6 clusters. To visualize clusters, we applied t‐SNE to our data points using two dimensions, a perplexity of 4 and a maximum of 1000 iterations. Basing our clustering analysis on data obtained from the high ASM concentrations was motivated by the reasoning that effects in this condition are likely stronger and more reliable. The hierarchical clustering approach was chosen as it allows representation of hierarchical relationships in the form of a dendrogram, which is particularly useful for small datasets.

### 2.5 Statistical analysis

Statistical analyses for the five selected parameters—mean firing rate, burst duration, burst frequency, network burst frequency, and synchrony index—were performed in Python (version 3.12.7) using the stats models (0.14.6) and Scipy (1.17.1) libraries. As parameters were not independent from each other, the significance threshold (*α* = .05) was adjusted using the Cheverud correction,^10^ which estimates the effective number of independent tests among correlated variables. For each ASM, concentration, and parameter, the baseline and wash‐in condition was subsequently compared using a two‐sided Wilcoxon signed‐rank test. To control the false discovery rate (FDR) among the multiple comparisons conducted on the 18 ASMs and two concentrations per ASM, *p*‐values were adjusted using the Benjamini–Hochberg (BH) procedure. Differences were considered statistically significant when the BH‐adjusted *p*‐value was less than .01713 (*), .00338 (**), and .00034 (***), corresponding to alpha levels of .05, .01, and .001 before the Cheverud correction, respectively. Statistics, effect size, and the number of experimental replicates are indicated in Table S2.

### 2.6 RNA extraction and RT‐qPCR

Messenger RNA (mRNA) levels were measured by reverse transcription‐quantitative polymerase chain reaction (RT‐qPCR) as described previously.^8^ RNA was prepared from primary cortical neuron culture using a RNeasy Micro Kit (74 034, Qiagen) according to the manufacturer's instructions. Total RNA (500 ng) was used as a template for reverse transcription using iScript Select cDNA Synthesis Kit (1 708 896, BioRad) following the manufacturer's instructions. Quantitative PCR was performed using Luna Universal qPCR Master Mix (M3003E, New England BioLabs) according to the manufacturer's instructions. Data were shown as the ratio of the mRNA level of the test gene to that of β‐actin. All primers are shown in Table S3.

## 3 RESULTS

We performed MEA recordings under conditions identical to those used in a previous study.^8^ Compared with our previous study, cultures were maintained in BME‐based medium, which promoted faster network maturation and more robust spontaneous activity. Primary cortical neurons were cultured on a 24‐well MEA plate (Figure 1A) and recorded every 2–3 days until stable and regular burst activity was established (Figure 1B). At early stage (DIV5‐12), neuronal activity was characterized primarily by isolated spikes and single‐electrode bursts (Figure 1C, green). As the cell culture matures (DIV15‐21), burst activity became synchronized across multiple electrodes, giving rise to network bursts (Figure 1C, orange). At this stage, ASMs were applied and neuronal activity was recorded using a standardized protocol consisting of baseline, wash‐in, and wash‐out (Figure 1B). Oxcarbazepine (OXC, 50 μM) is shown as a representative example demonstrating robust suppression of network activity during drug application (Figure 1C). Representative examples illustrate the definitions of individual spikes, bursts, as well as network bursts used in the study (Figure 1D, green: single‐electrode bursting, orange: network burst). For each ASM, test concentrations were selected based on the indicators of clinically relevant concentrations (Table S4), and two concentrations were evaluated.

**FIGURE 1:**
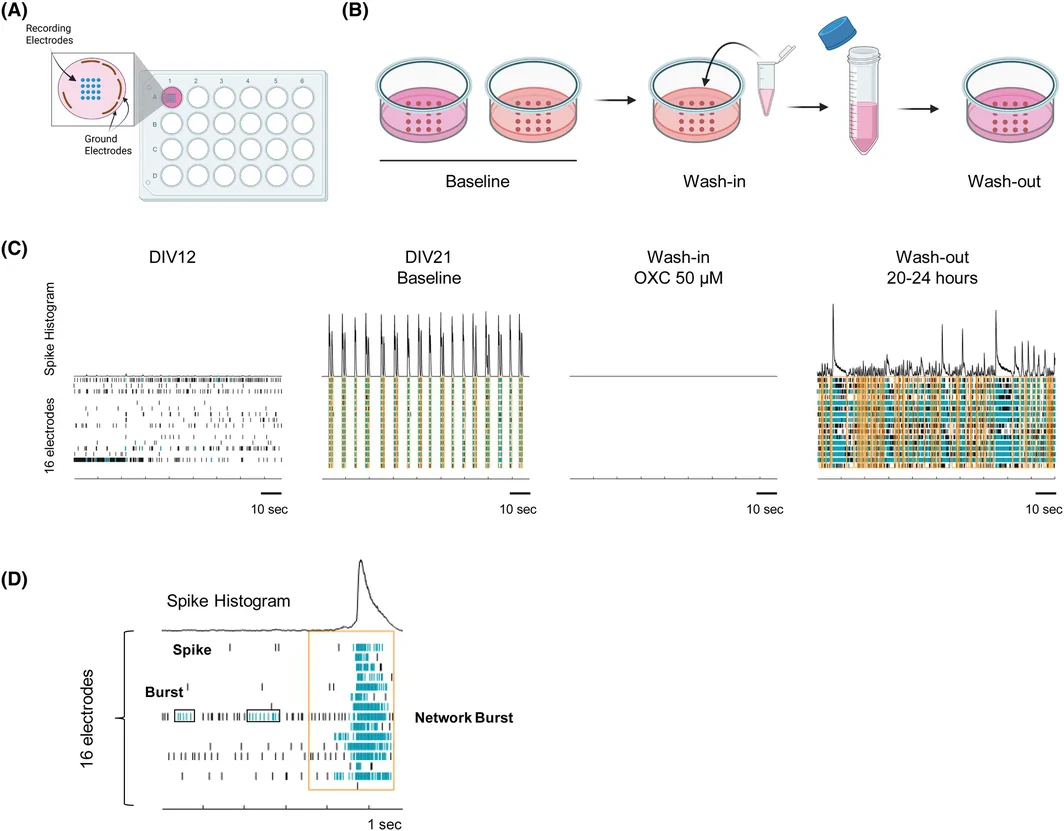
Multielectrode array (MEA) protocol. (A) Primary cortical neurons were cultured in 24‐well MEA plates. (B) Baseline of neurons was recorded every 2–3 days until drug application at days in vitro (DIV) 15–25. Wash‐out recording was conducted 20–24 h after wash‐in. (C) Representative spike histograms (the top half) and signal traces (the bottom half) from a selected well with 16 electrodes at baseline, during wash‐in (oxcarbazepine, OXC 50 μM), and at wash‐out. The spike histogram (the top half of the diagram) represents the total number of spikes detected across 16 electrodes in a well (24‐well plate). (Green: single‐electrode bursting, orange: Network burst). (D) In the signal traces, spikes were dected when the signal crossed the threshold of 6x standard deviation of the baseline. A cluster of spikes (5 spikes less than 100ms) was defined as a burst, and when it was coordinated across multiple electrodes, it was termed a network burst. (green: single‐electrode bursting, orange: network burst).

MEA recordings initially yielded 44 parameters, describing spiking, bursting, network bursting, and network synchrony. To evaluate parameter redundancy, pairwise Pearson correlation coefficients were calculated for all parameters, revealing extensive interdependence (Figure S1A). PCA showed that the first principal component (PC1) explained more than 60% of the total variance (Figure S1B). Of note, loadings for PC1 were distributed broadly across parameters, indicating that no individual variable disproportionately contributed to the observed differences among ASMs (Figure S1C). Because the present study employed a low‐density MEA platform, analyses were focused on the principal activity domains that can be robustly and reproducibly quantified in this preparation: spiking, bursting, network bursting, and synchrony. From these domains, five representative parameters were selected. Mean firing rate was chosen to quantify overall neuronal excitability, burst frequency, and burst duration to characterize local bursting behavior, network burst frequency to assess coordinated population activity, and synchrony index to quantify temporal coupling across electrodes. These parameters collectively provide a biologically interpretable summary of neuronal network function while avoiding overrepresentation of highly correlated variables. The pairwise Pearson correlation coefficients among the five selected parameters are displayed in Figure 2A.

**FIGURE 2:**
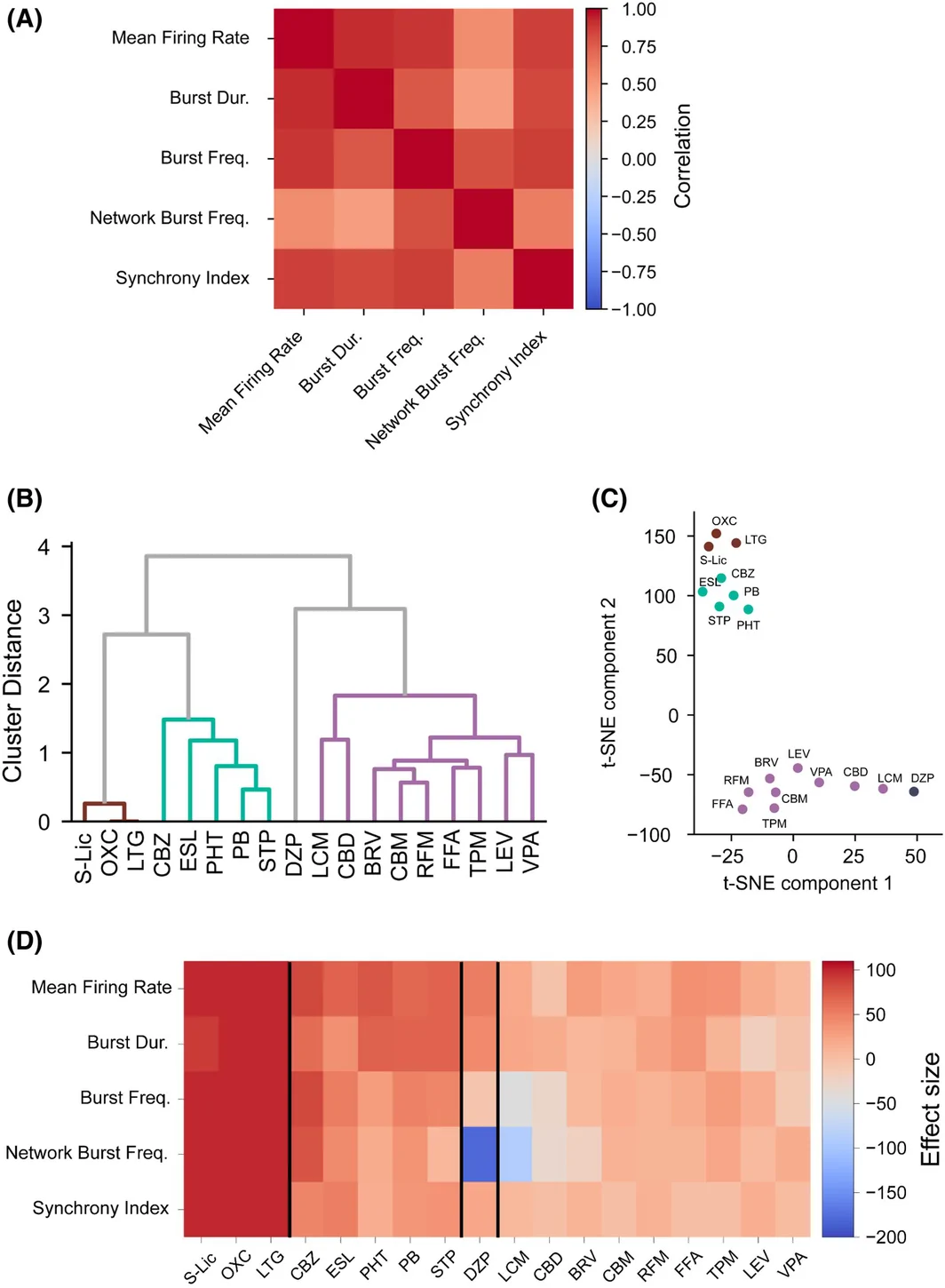
Effect similarity among 18 anti‐seizure medications (ASMs). (A) Heatmap displaying pairwise Pearson correlation coefficients among five representative parameters. Correlations are based on the average effect size for the high‐concentration condition of each drug. For the full correlation matrix including all 44 parameters refer to Figure S1A. (B) Dendrogram displaying the four clusters of ASMs (high concentration) obtained from hierarchical clustering. Clustering was based on the five representative parameters displayed in A. (C) t‐SNE plot visualizing the two‐dimensional data points obtained for the different drugs. Each point represents one ASM (using the high concentration) and is colored according to its cluster membership. (D) Heatmap displaying the effect of 18 ASMs (high concentration) on the five representative parameters that were used for clustering. Drugs are ordered by their cluster membership with vertical black lines separating clusters.

We next grouped the 18 tested ASMs according to their effect on these five parameters. Hierarchical clustering identified four clusters, corresponding to the highest silhouette score obtained across the tested range of clusters (silhouette score = .54, Figure 2B). Visualization of the data using t‐SNE revealed a cluster structure consistent with the hierarchical clustering results (Figure 2C). Differences between clusters were further illustrated by a heatmap displaying the average effect size for each ASM across five representative parameters (Figure 2D).

To illustrate the characteristic activity profiles of the four clusters, representative compounds were selected from each group (Cluster 1: S‐Lic, OXC; Cluster 2: STP; Cluster 3: DZP; Cluster 4: BRV and LEV). Cluster 1 compounds produced the strongest effects on neuronal network activity. S‐Lic (250 μM) and OXC (50 μM) significantly reduced all five parameters and nearly abolished network activity (Figure 3A,C–G). In contrast, Cluster 2 compounds exhibited a more selective profile. For instance, STP (100 μM) affected mean firing rate, burst duration, and synchrony, while exerting comparatively modest effects on burst frequency and network burst frequency (Figure 3C–G). DZP (1 μM), the solely member of Cluster 3, produced only limited effects across all five parameters (Figure 3C–G). Similarly, BRV (50 μM) and LEV (300 μM), representative compounds of Cluster 4, induced only minor changes in neuronal network activity (Figure 3B–G).

**FIGURE 3:**
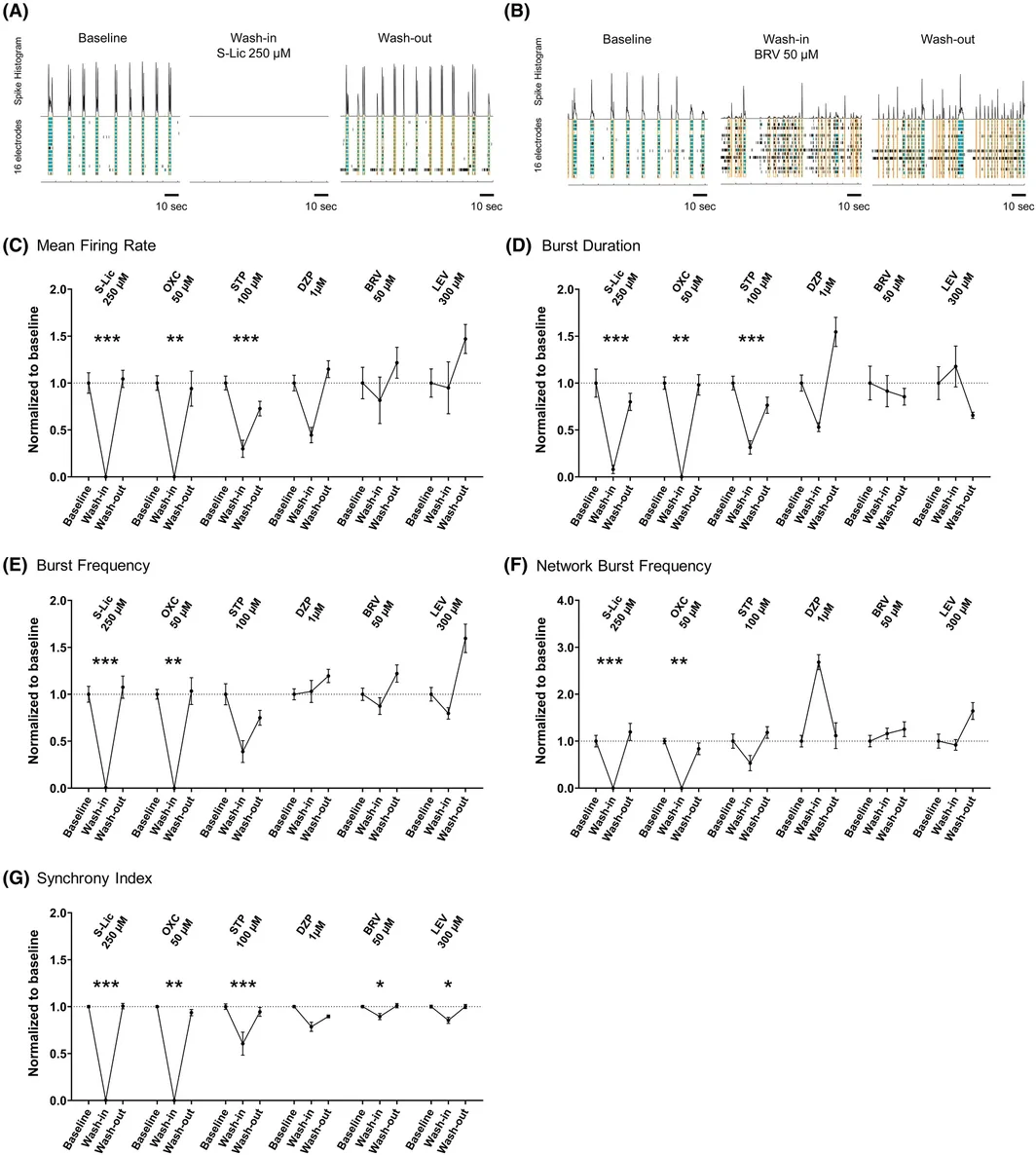
Effects of anti‐seizure medications representing each cluster on cortical neuronal network activities. (A, B) Representative spike histograms and signal traces from a selected well with 16 electrodes at baseline, during wash‐in (A. S‐Lic 250 μM; B. BRV 50 μM), and at wash‐out. (C–G) All activities were normalized to DMSO administration, and further normalized to baseline. (C) Mean firing rate. (D) Average burst duration. (E) Average burst frequency. (F) Average network burst frequency. (G) Synchrony index. Differences were considered statistically significant when the BH‐adjusted *p*‐value was less than .01713 (*), .00338 (**), and .00034 (***), corresponding to alpha levels of .05, .01, and .001 before the Cheverud correction, respectively.

To facilitate systematic comparison across compounds, effect sizes for all five parameters were visualized using radar plots (Figure 4). Raw data are provided in Table S1, and statistics analyses and effect sizes are summarized in Table S2. Differences were considered statistically significant when the BH‐adjusted *p*‐value was less than .01713 (*), .00338 (**), and .00034 (***), corresponding to alpha levels of .05, .01, and .001 before the Cheverud correction, respectively. Cluster 1 corresponded to Na^+^ channel blockers (S‐Lic, OXC, LTG), characterized by broad suppression of both single‐cell and network activity (Figure 4A). Cluster 2 included Na^+^ channel blockers (CBZ, ESL, PHT) along with enhancers of GABAergic inhibition (PB, STP), which influenced spike and burst duration, with little to no effect on burst frequency (PHT, PB, STP) or network bursting (ESL, PHT, PB, STP) or synchrony (CBZ, PB) (Figure 4B). Cluster 3 contained only a single member, DZP, which remains challenging to interpret within this experimental framework (Figure 4C). The remainder of the ASMs was assigned to Cluster 4 and generally produced only modest measurable effects under the present experimental conditions (Figure 4D). Together, these findings demonstrate that MEA‐based network analysis combined with multivariate clustering can resolve distinct functional signatures among clinically used ASMs.

**FIGURE 4:**
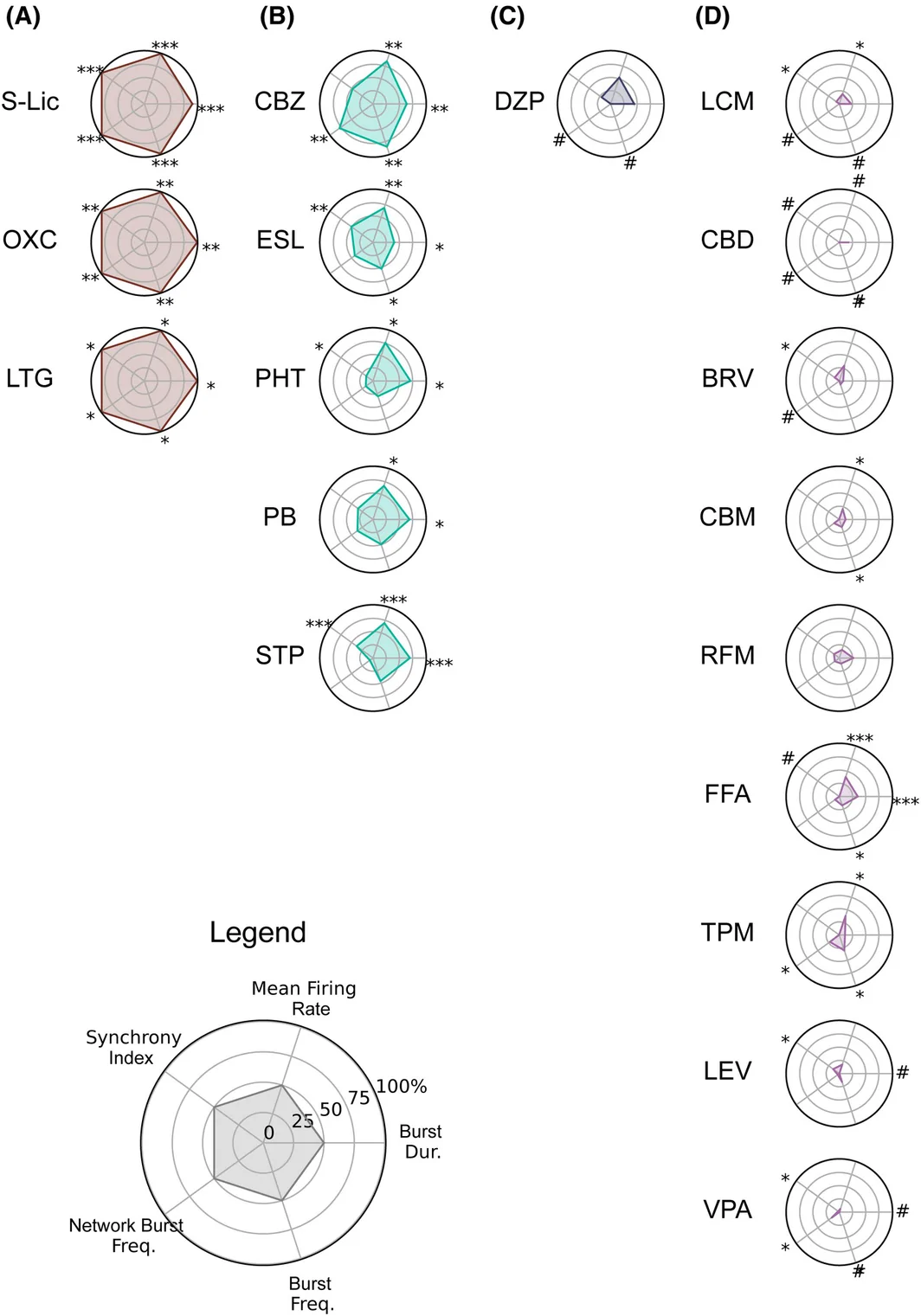
A radar chart of 18 ASMs classified into four clusters with five parameters. (A) Cluster 1. (B) Cluster 2. (C) Cluster 3. (D) Cluster 4. The effect size of ASM is the change in activity, determined by subtracting the remaining activity from 100%, with each parameter represented as one axis on the radar chart. Differences were considered statistically significant when the BH‐adjusted *p*‐value was less than .01713 (*), .00338 (**), and .00034 (***), corresponding to alpha levels of .05, .01, and .001 before the Cheverud correction, respectively. ^#^The effect size was negative (see Figure 3F, DZP). These effect sizes were obtained with high drug concentration.

## 4 DISCUSSION

In the present study, we evaluated the effects of 18 ASMs on neuronal network activity using primary neuron cultures and MEA recordings. By combining MEA‐derived parameters with multivariate analysis and unsupervised clustering approaches, we classified ASMs into four distinct functional clusters. This classification partially corresponded to conventional pharmacological categories.

MEA recordings provide a valuable intermediate approach between single‐cell electrophysiology and in vivo models. Unlike patch‐clamp recordings, which provide detailed information at the cellular level but are limited in throughput, MEA systems enable simultaneous and long‐term, cost‐effective monitoring of neuronal populations under highly standardized conditions. In addition, MEA‐based assays substantially reduce animal use compared with many in vivo screening approaches. Although these advantages have prompted a widespread use of MEA‐based platforms for neurotoxicity testing and evaluation of individual ASMs,^5^, ^6^, ^7^, ^11^ systematic comparison of a large panel of clinically relevant ASMs under identical experimental conditions has remained limited. Our study demonstrates that MEA recordings combined with multivariate analysis can generate reproducible pharmacological signatures that facilitate direct comparison across compounds.

A key aspect of the present study was the reduction of a large set of MEA‐derived variables into a smaller group of representative parameters. Correlation and PCA analyses revealed substantial redundancy among the recorded parameters, with no single variable dominating the observed variation. We therefore selected five biologically interpretable parameters representing the principal activity domains measurable with low‐density MEA recording: spiking, bursting, network bursting, and synchrony. Hierarchical clustering based on these parameters identified four ASM clusters and provided a compact representation of network‐level drug effects.

The resulting four clusters showed partial, but not complete, correspondence with known mechanisms of action. Cluster 1 contained Na^+^ channel blockers (S‐Lic, OXC, LTG), all of which produced robust suppression of both single‐cell and network‐level activity. The strong effects of S‐Lic and OXC are consistent with their potent modulation of voltage‐gated sodium channels and their ability to suppress sustained neuronal firing. Of interest, LTG exhibited a similar network profile despite its distinct chemical structure, suggesting convergence at the level of network function rather than molecular target specificity.

Cluster 2 comprised both Na^+^ channel blockers (CBZ, ESL, PHT) and enhancers of GABAergic inhibition (PB, STP). In contrast to Cluster 1, these compounds primarily affected firing and burst duration while exerting comparatively smaller effects on burst frequency and network burst frequency and synchrony. Thus, in this case, despite the distinct molecular targets (sodium channels vs GABA receptors) the functional signatures at the network level are similar. Of interest, CBZ and ESL share both chemical similarity with the Cluster 1 compounds S‐Lic and OXC, all being derived from the dibenzazepine scaffold. All four compounds are sodium channel blockers, yet they are segregated into different clusters. This underscores that network effects may not be directly predictable from the mechanism of action or chemical similarity. More generally, the clustering results illustrate that network activity may reflect the integrated outcome of subtle differences in pharmacological effects, rather than the identity of molecular targets alone.

For Cluster 3 (comprising only DZP) and Cluster 4, comprising FFA (serotonin modulator^12^, ^13^), TPM (multiple mechanisms of action), and BRV and LEV (SV2A selective ligands^14^), and LCM (selectively enhances slow inactivation of Na channels^15^), and ASMs with complex or incompletely understood mechanisms (CBD,^16^ RFM,^17^ and VPA^18^), we found minimal detectable effects under our conditions. These finding should be interpreted cautiously. First, the primary cortical neuron cultures used in this study may not fully express all relevant drug targets. Consistent with this possibility, RT‐qPCR analysis indicated incomplete expression of several target genes (Figure S2). Second, only acute drug effects were examined. Some ASMs may require prolonged exposure, more mature neuronal networks, or pathological activity states to reveal their full pharmacological actions. Third, the present experiments were performed in spontaneously active, non‐epileptic neuronal cultures. Consequently, drugs that primarily suppress epileptiform activity may show limited effects in this preparation despite robust clinical efficacy.

The study has several additional limitations. Primary neuron cultures do not reproduce the cellular diversity, connectivity, and pathological alterations present in epileptic brain networks. Furthermore, the 16‐electrode MEA platform used here is optimized for analysis of spikes, bursts, and synchrony but does not permit detailed investigation of local field potentials or oscillatory network activity. Future studies using epileptiform activity models, chronic drug exposure paradigms, or high‐density MEA systems may provide additional insight into ASM‐specific network effects.

Despite these limitations, the present study demonstrates that MEA recordings with primary neuron cultures provide a robust and scalable platform for systematically comparing ASMs under standardized conditions. Moreover, by applying multivariate and unsupervised clustering approaches, we were able to classify drug‐induced network signatures independently of their conventional mechanisms of action. These findings enable identification of characteristic network activity patterns associated with specific ASMs and offers a complementary strategy to single‐cell electrophysiology. By enabling direct, head‐to‐head comparison across a broad range of compounds, this framework may serve a useful foundation for future pharmacological screening and drug discovery efforts.

## AUTHOR CONTRIBUTIONS

Griselda Marku: Methodology, investigation, participation of making of the figures, and data curation. Lena Gschossmann: Methodology, formal analysis, realization of figures, and participation in writing of the manuscript. Kerstin Hallmann: Methodology, investigation, and data curation. Heinz Beck: Conception of the study, manuscript writing, review and editing, supervision, and funding acquisition. Kunihiko Araki: Conception and design of the study, investigation, data curation, manuscript writing, review and editing, realization of figures and tables, supervision, and project administration. All authors contributed to the article and approved the submitted version.

## FUNDING INFORMATION

Supported by BMFTR (Federal Ministry of Research, Technology and Space) Program TREATION (Treat‐ION), New Therapies for Neurological Ion Channel and Transporter Disorders (Project P5) to H.B. and Go‐Bio initial Project ‘EpiTher’ of the BMFTR to H.B. and K.A.

## CONFLICT OF INTEREST STATEMENT

Heinz Beck was a consultant for BIAL.

## ETHICS STATEMENT

We confirm that we have read the Journal's position on issues involved in ethical publication and affirm that this report is consistent with those guidelines.

## Supporting information


**Figure S1.** (A) Pairwise Pearson correlation coefficients among 44 parameters derived from multi‐electrode array (MEA) recordings. Correlations are based on the average effect size for the high‐concentration condition of each drug. (B) Principal component analysis (PCA) performed on 43 parameters (one parameter had to be removed due to a missing value) indicated that more than 60% of variance were explained by the first principal component (PC1) alone. (Expl. Variance = Explained Variance) (C) Individual parameters contributed similarly to the PC1 as indicated by a relatively uniform distribution of weights (sort order and color represent parameter ranking by PC1 weight).

**Figure S2.** Relative mRNA expression level in primary cortical neuron cultures at 4 weeks. The results of Scn2a* and Scn8a* were cited from our previous paper^8^ as references.

**Table S1.** All raw data of multi‐electrode array (MEA) recordings.

**Table S2.** All statistics of multi‐electrode array (MEA) recordings.

**Table S3.** Primer list.
**Table S4**. Anti‐seizure medication list with concentration.

## Data Availability

All raw data are provided in Table S1. The data are available from the corresponding author upon reasonable request.
