# Supplementary material for: Acute effects of anti‐seizure medications on network level dynamics: A systematic comparison using multielectrode arrays
Source: Epilepsia. 2026 Aug 1;67(11):6108–18. doi: 10.1002/epi.70408 (PMC13768903; doi:10.1002/epi.70408)
Supplement: Supplementary file 1 [file EPI-67-6108-s005.docx]

**Supp. Fig1.** (**A**) Pairwise Pearson correlation coefficients among 44 parameters derived from MEA recordings. Correlations are based on the average effect size for the high-concentration condition of each drug. (**B**) Principal component analysis (PCA) performed on 43 parameters (one parameter had to be removed due to a missing value) indicated that more than 60 % of variance were explained by the first principal component (PC1) alone. (**C**) Individual parameters contributed similarly to the PC1 as indicated by a relatively uniform distribution of weights (sort order and color represent parameter ranking by PC1 weight).

**Supp. Fig 2. Relative mRNA expression level in primary cortical neuron cultures at 4 weeks.**

The results of Scn2a* and Scn8a* were cited from our previous paper^8^ as references.

**Supp. Table 1. All raw data of MEA recordings.**

**Supp. Table 2. All statistics of MEA recordings.**

**Supp. Table 3. Primer list.**

| No | Name | Forward | Reverse |
| --- | --- | --- | --- |
| 1 | Scn2a | GGTCATGGTGATTGGGAACCTTG | CACGGCTATCTGGAGGTTGTTC |
| 2 | Scn8a | GGGAGGACGATGAAGACAG | TCAGGCAGAACACCGTCAG |
| 3 | β Actin | GGCTACAGTTTCACCACCAC | ACTCCTGCTTGCTGATCCAC |
| 4 | Cacna1h | GAACGTGGTTCTTTACAACGGC | GCACATAGTTCCCAAAGGTCA |
| 5 | Gabra1 | CACCATGAGGTTGACCGTGA | TACAACCACTGAACGGGCTG |
| 6 | Gad1 | TGATCGCTCCACCAAGGTTC | ATGAGAACAAACACGGGTGC |
| 7 | Gria2 | AGGACTACGGCAGAAGGAGT | CCAGCATTGCCAAACCAAGG |
| 8 | Sv2a | GTCTTTGTGGTGGGCTTTGT | CGAAGACGCTGTTGACTGAG |
| 9 | Htr1a | TGCCAACTATCTCATCGGCTCC | CAGAGTCCACTTGTTGAGCACC |

**Supp. Table 4. ASMs list with concentration**

| No | Name | Abbreviation | Molecular Weight (g/mol) | Indicators of clinical concentration | | Present study | Reference |
| --- | --- | --- | --- | --- | --- | --- | --- |
|  |  |  |  | µg/mL or ng/mL | µM or nM | mM or µM |  |
| 1 | Carbamazepine | CBZ | 236 | 2 - 18 µg/mL | 8.5 - 76 µM | 25 µM, 50 µM | ^1, 2^ |
| 2 | Oxcarbazepine | OXC | 252 | 8.5 µg/mL | 33.7 µM | 25 µM, 50 µM | ^3^ |
| 3 | Eslicarbazepine acetate | ESL | 296 | 3 - 25 µg/mL (S-Lic) | 11.8 - 98.4 µM | 25 µM, 50 µM | ^4^ |
| 4 | S-licarbazepine | S-Lic | 254 | 3 - 25 µg/mL | 11.8 - 98.4 µM | 50 µM, 250 µM | ^4^ |
| 5 | Lacosamide | LCM | 250 | 2 - 10 µg/mL | 8 - 40 µM | 50 µM, 100 µM | ^5^ |
| 6 | Phenytoin | PHT | 252 | 10 - 20 µg/mL | 39.6 - 79.3 µM | 50 µM, 100 µM | ^6^ |
| 7 | Cenobamate | CBM | 267 | 5 - 35 µg/mL | 18.7 - 131 µM | 50 µM, 100 µM | ^7^ |
| 8 | Lamotrigine | LTG | 256 | 2.5 - 10 µg/mL | 9.76 - 39 µM | 25 µM, 100 µM | ^8, 9^ |
| 9 | Cannabidiol | CBD | 314 | IV: 686 ng/mL | IV: 2.18 µM | 2.5 µM, 5 µM | ^10^ |
| 10 | Levetiracetam | LEV | 170 | 12 - 46 µg/mL | 70.5 - 270 µM | 100 µM, 300 µM | ^11^ |
| 11 | Brivaracetam | BRV | 212 | 1.95 µg/mL | 9.2 µM | 10 µM, 50 µM | ^12^ |
| 12 | Stiripentol | STP | 234 | 14 - 33 µg/mL | 59.8 - 141 µM | 50 µM, 100 µM | ^13^ |
| 13 | Fenfluramine | FFA | 231 | 41.4 ng/mL | 179 nM | 5 µM, 20 µM | ^14^ |
| 14 | Rufinamide | RFM | 238 | 3.8 - 7.7 µg/mL | 15.9 - 32.3 µM | 50 µM, 100 µM | ^15^ |
| 15 | Valproic acid | VPA | 144 | 34 µg/mL | 236 µM | 0.5 mM, 1 mM | ^16^ |
| 16 | Topiramate | TPM | 339 | 5.5 µg/mL | 16.2 µM | 25 µM, 100 µM | ^17^ |
| 17 | Phenobarbital | PB | 232 | 13.1 µg/mL | 56.4 µM | 25 µM, 50 µM | ^18^ |
| 18 | Diazepam | DZP | 284 | IV: 400 ng/mL | IV: 1.4 µM | 0.3 µM, 1 µM | ^19^ |

**■References**

1.	Marino SE, Birnbaum AK, Leppik IE, Conway JM, Musib LC, Brundage RC, et al. Steady-state carbamazepine pharmacokinetics following oral and stable-labeled intravenous administration in epilepsy patients: effects of race and sex Clin Pharmacol Ther. 2012 Mar;91:483-488.

2.	Garnett WR, Levy B, McLean AM, Zhang Y, Couch RA, Rudnic EM, et al. Pharmacokinetic evaluation of twice-daily extended-release carbamazepine (CBZ) and four-times-daily immediate-release CBZ in patients with epilepsy Epilepsia. 1998 Mar;39:274-279.

3.	Lloyd P, Flesch G, Dieterle W. Clinical pharmacology and pharmacokinetics of oxcarbazepine Epilepsia. 1994;35 Suppl 3:S10-13.

4.	Singh RP, Asconape JJ. A review of eslicarbazepine acetate for the adjunctive treatment of partial-onset epilepsy J Cent Nerv Syst Dis. 2011;3:179-187.

5.	Iwasaki T, Kobayashi T, Miyamoto Y, Imaizumi T, Kaku S, Udagawa N, et al. Efficacy and Blood Levels of Lacosamide in Patients with Focal Epilepsy J Clin Med. 2024 Nov 19;13.

6.	Kozer E, Parvez S, Minassian BA, Kobayashi J, Verjee Z, Koren G. How high can we go with phenytoin? Ther Drug Monit. 2002 Jun;24:386-389.

7.	Steinhoff BJ, Georgiou D, Dietmann D, Intravooth T. Cenobamate Plasma Levels in Patients with Epilepsy: Correlation with Efficacy and Tolerability? J Clin Med. 2024 May 8;13.

8.	Chen C, Casale EJ, Duncan B, Culverhouse EH, Gilman J. Pharmacokinetics of lamotrigine in children in the absence of other antiepileptic drugs Pharmacotherapy. 1999 Apr;19:437-441.

9.	Lee ZN, van Nuland M, Bognar T, Leijten FSS, van der Elst KCM. Association of Lamotrigine Plasma Concentrations With Efficacy and Toxicity in Patients With Epilepsy: A Retrospective Study Ther Drug Monit. 2024 Oct 1;46:642-648.

10.	Crockett J, Critchley D, Tayo B, Berwaerts J, Morrison G. A phase 1, randomized, pharmacokinetic trial of the effect of different meal compositions, whole milk, and alcohol on cannabidiol exposure and safety in healthy subjects Epilepsia. 2020 Feb;61:267-277.

11.	Shi M, Liu C, He L, Wu H, Wu Y. Therapeutic drug monitoring and the therapeutic reference range of levetiracetam for Chinese patients: Problems and issues Seizure. 2023 Jul;109:26-33.

12.	Hentschel M, Stoffel-Wagner B, Surges R, von Wrede R, Dolscheid-Pommerich RC. Value of drug level concentrations of brivaracetam, lacosamide, and perampanel in care of people with epilepsy Epilepsia. 2024 Mar;65:620-629.

13.	Wheless J, Weatherspoon S. Use of Stiripentol in Dravet Syndrome: A Guide for Clinicians Pediatr Neurol. 2025 Jan;162:76-86.

14.	Schoonjans AS, Roosens L, Dewals W, Paelinck BP, Ceulemans B. Therapeutic drug monitoring of fenfluramine in clinical practice: Pharmacokinetic variability and impact of concomitant antiseizure medications Epilepsia. 2022 Mar;63:686-696.

15.	Perucca E, Cloyd J, Critchley D, Fuseau E. Rufinamide: clinical pharmacokinetics and concentration-response relationships in patients with epilepsy Epilepsia. 2008 Jul;49:1123-1141.

16.	Pollack GM, McHugh WB, Gengo FM, Ermer JC, Shen DD. Accumulation and washout kinetics of valproic acid and its active metabolites J Clin Pharmacol. 1986 Nov-Dec;26:668-676.

17.	Glauser TA, Miles MV, Tang P, Clark P, McGee K, Doose DR. Topiramate pharmacokinetics in infants Epilepsia. 1999 Jun;40:788-791.

18.	Browne TR, Evans JE, Szabo GK, Evans BA, Greenblatt DJ. Studies with stable isotopes II: Phenobarbital pharmacokinetics during monotherapy J Clin Pharmacol. 1985 Jan-Feb;25:51-58.

19.	Friedman H, Greenblatt DJ, Peters GR, Metzler CM, Charlton MD, Harmatz JS, et al. Pharmacokinetics and pharmacodynamics of oral diazepam: effect of dose, plasma concentration, and time Clin Pharmacol Ther. 1992 Aug;52:139-150.
